# What evidence exists on the ecological and physical effects of built structures in shallow, tropical coral reefs? A systematic map protocol

**DOI:** 10.1186/s13750-023-00313-2

**Published:** 2023-09-15

**Authors:** Avery B. Paxton, Todd M. Swannack, Candice D. Piercy, Safra Altman, Leanne Poussard, Brandon J. Puckett, Curt D. Storlazzi, T. Shay Viehman

**Affiliations:** 1 National Centers for Coastal Ocean Science, National Ocean Service, National Oceanic and Atmospheric Administration, 101 Pivers Island Road, Beaufort, NC 28516 USA; 2 U.S. Army Engineer Research and Development Center, 3909 Halls Ferry Road, Vicksburg, MS 39180 USA; 3 U.S. Geological Survey, Pacific Coastal and Marine Science Center, 2885 Mission Street, Santa Cruz, CA 95060 USA

**Keywords:** Artificial structures, Designed habitat, Coastal protection, Coastal restoration, Coastal mitigation, Eco-engineering, Natural infrastructure, Nature-based solutions, Nature-inspired designs

## Abstract

**Background:**

Shallow, tropical coral reefs face compounding threats from habitat degradation due to coastal development and pollution, impacts from storms and sea-level rise, and pulse disturbances like blast fishing, mining, dredging, and ship groundings that reduce coral reefs’ height and variability. One approach toward restoring coral reef structure from these threats is deploying built structures. Built structures range from engineered modules and repurposed materials to underwater sculptures and intentionally placed natural rocks. Restoration practitioners and coastal managers increasingly consider incorporating built structures, including nature-based solutions, into coral reef-related applications. Yet, synthesized evidence on the ecological and physical performance of built structure interventions across a variety of contexts (e.g., restoration, coastal protection, mitigation, tourism) is not readily available to guide decisions. To help inform management decisions, here we aim to document the global evidence base on the ecological and physical performance of built structures in shallow (≤ 30 m) tropical (35° N to 35° S latitude) coral ecosystems. The collated evidence base on use cases and associated ecological and physical outcomes of built structure interventions can help inform future consideration of built structures in reef restoration design, siting, and implementation.

**Method:**

To discover evidence on the performance of built structures in coral reef-related applications, such as restoration, mitigation, and coastal protection, primary literature will be searched across indexing platforms, bibliographic databases, open discovery citation indexes, a web-based search engine, a novel literature discovery tool, and organizational websites. The geographic scope of the search is global, and there is no limitation to temporal scope. Primary literature will be screened first at the level of title and abstract and then at the full text level against defined eligibility criteria for the population, intervention, study type, and outcomes of interest. Metadata will be extracted from studies that pass both screening levels. The resulting data will be analyzed to determine the distribution and abundance of evidence. Results will be made publicly available and reported in a systematic map that includes a narrative description, identifies evidence clusters and gaps, and outlines future research directions on the use of built structures in coral reef-related applications.

**Supplementary Information:**

The online version contains supplementary material available at 10.1186/s13750-023-00313-2.

## Background

Coral reefs provide extensive ecosystem services, including biodiversity benefits, coastal protection, and fisheries provisioning [53], yet face global declines from multiple threats [15, 28]. Local threats include those from habitat degradation often linked to coastal development [24], overfishing [54], and pollution [32, 34, 55], as well as from disturbances like blast fishing [52], coral mining [10], dredging [17], and ship groundings [37]. Global stressors from climate change include mortality from ocean warming and associated bleaching [29], disease [27], and ocean acidification [12]. Climate change is also increasing the severity and frequency of storms that can further degrade coral reefs by breaking and dislodging coral [18] and increasing sedimentation, which reduces the potential for successful coral recruitment [16, 47].

Strategies to slow or reverse declines in coral reefs often include restoration, such as direct transplantation of corals or larval enhancement [5]. Coastal managers and restoration practitioners are considering the incorporation of built structures into coral restoration design and implementation [50]. Here, we define built structures as those that have been engineered, designed, created, built, or constructed using artificial, hybrid, or natural materials. We define restoration broadly, following the UN Decade on Ecosystem Restoration, as “efforts to prevent, halt, or reverse the degradation of ecosystems” [48]. For coral reefs, this definition includes partial or holistic ecosystem recovery and thus actions aimed towards returning reefs to a historical state or creating new reefs [49, 50]. Built structures have a centuries-long history of being deployed in the seascape for multiple objectives. For example, artificial reefs have been purposely sunk since the 1600 s [44] to increase fishing yield, provide recreation opportunities, and conduct scientific research experiments, but in select cases have also been used expressly to restore coral reefs by creating, replacing, supplementing, enhancing, or stabilizing structured habitat [4, 25]. These intentionally deployed structures include those that have been repurposed from their original uses (e.g., concrete pipes originally used in construction), as well as modules designed for particular contexts, such as reef restoration (e.g., target species, environmental settings) [31, 43]. In the past decade, underwater artwork installations have grown in popularity, as artwork and sculpture gardens have been commissioned and implemented to help restore corals and generate locations for recreational divers to enjoy [3, 41] (1000 Mermaids Artificial Reef Project, https://1000mermaids.com/).

Built structures have also been used for environmental mitigation and coastal protection purposes. Structures installed for environmental mitigation seek to address impacts from disturbances like blast fishing, ship grounding, coral mining, dredging, and storms, which can reduce reef height and complexity and create excess amounts of rubble that prevent survival of coral recruits [8, 51]. In these instances, deployment of natural rock, hybrid structures (e.g., rock with cement, rock with mesh net), or human-made structures (e.g., concrete) can help stabilize rubble and allow for recruit survival [8]. The role of coral reefs in providing coastal protection benefits has become increasingly apparent as coral reefs can reduce wave energy by ~ 97% where present [19] and thus provide ~ $1.8 billion in hazard risk reduction benefits per year in the U.S. alone [39]. In response to the risk reduction benefits, new initiatives have been launched to design engineered reefs for coastal protection. In Grenada, for example, modular engineered structures were deployed to help reduce coastal erosion and flooding [38], while in southeast India, trapezoidal artificial modules were deployed to dissipate wave energy [30]. Newly funded Department of Defense projects in the U.S. aim to create hybrid reef structures that incorporate some “gray” engineering (e.g., traditional hardened structures) and some “green” (e.g., natural components and nature-based solutions) ecological objectives to mitigate flooding, erosion, and storm damage (Reefense, https://www.darpa.mil/program/reefense).

Despite the history and increasing consideration of built structures for coral restoration and related applications like environmental mitigation and coastal protection, questions remain regarding how built structures should be considered in management and restoration decisions. Central to these questions is that the global evidence base regarding the use and performance of built structures has not been collated or synthesized; but, see syntheses for particular contexts, such as artificial reefs [25], substrate stabilization [8], and 3D technology for reef structures [33]. The lack of broadly synthesized evidence presents barriers to implementing management and policy decisions regarding future use of built structures in coral reef systems. Without synthesized evidence, it is challenging for decision makers to rigorously and reproducibly evaluate whether built structures may be appropriate tools in particular environmental settings and use-case scenarios.

The goal of this study is to collate evidence on the ecological and physical performance of built structure interventions in shallow, tropical coral reef settings. This synthesis of knowledge will help inform practice for built structure design and implementation, including as nature-based solutions that can help address societal and ecological challenges. Because built structures have been used for multiple applications related to tropical coral reefs, such as for restoration, coastal protection and environmental mitigation, we will include evidence from these diverse bodies of literature. This will ensure that our synthesis stems from the most comprehensive body of relevant literature and will also help ensure that findings from our synthesis can be used to help guide management decisions regarding the design, siting, and implementation of gray-green infrastructure in coral reef settings.

### Stakeholder engagement

This project was jointly conceptualized by scientists from the National Oceanic and Atmospheric Administration (NOAA) National Centers for Coastal Ocean Science (NCCOS), the U.S. Army Corps of Engineers (USACE) Engineering with Nature (EWN) Program, and the U.S. Geological Survey (USGS) Coastal and Marine Hazards and Resources Program (CMHRP) to synthesize how built structures have been used in a variety of contexts, such as those related to coral restoration, coastal protection, and environmental mitigation. The motivation for the synthesis was to catalog uses of and ecological and physical performance outcomes associated with built structures in shallow, tropical coral reef settings to help inform hybrid or gray-green reef structure design, siting, implementation, and potentially policy decisions. The core team of scientists from NOAA, USACE, and USGS scoped the systematic map and developed the search strategy based on stakeholder needs. Because the core team does not include international scientists, additional stakeholders will be consulted during the development of the systematic map to help ensure that primary literature from international sources is captured by the systematic map.

## Objective of the systematic map

The objective of this systematic map is to document the global evidence base on the performance (ecological and physical) of built structures in shallow, tropical coral reef settings. The systematic map also aims to summarize how evidence differs by built structure qualities, such as the type and material of intervention, as well as the goal and seascape setting.

Question: What is the distribution and abundance of evidence on the ecological and physical performance of built structures in shallow, tropical coral reef systems?

Sub-questions:

- How does the distribution and abundance of evidence on the performance of built structures used in coral reef-related applications differ by intervention type (e.g., human-made—designed or engineered structures, human-made repurposed structures, human-made artwork, hybrid structures of artificial and natural origin, and natural structures of geologic origin)?
- For which types (e.g., reef modules, concrete pipes, natural rock, mesh over rubble) and materials (e.g., concrete, metal, rock, fiberglass) of built structures has the performance been evaluated?
- For which ecological and physical outcomes has the performance of built structures used in coral reef-related applications been evaluated?
- How does the distribution and abundance of evidence on built structures differ by intervention goal or context (e.g., restoration, environmental mitigation, coastal protection, tourism), seascape setting (e.g., depth, energetic environment, relative location on reef), spatial scale, and geographic region?

Elements of the primary question: Elements of the primary question are the population, intervention, comparator, outcome, and study type (Table 1).

**Table 1:** Summary of elements of the primary question, including population, intervention, comparator, outcome, and study type

Population	Coral reefs located in shallow, tropical coastal environments (≤ 30 m, 35° N to 35° S latitude)
Intervention	Built structures of human-made, hybrid, or natural origin established in coral systems
Comparator	No comparator required beyond presence of built structure intervention. Studies that include a comparator (presence vs. absence of built structure intervention, before vs. after built structure intervention, different types of built structure interventions, etc.) will also be included
Outcome	Ecological (coral related) or physical (e.g., waves, current, flooding) performance outcomes associated with built structure intervention
Study type	Experimental, quasi-experimental, observational, or modeling studies with quantitative data on ecological or physical outcomes associated with the intervention. Studies can be conducted in the field or lab settings

## Methods

The systematic map will follow evidence synthesis standards from the Collaboration for Environmental Evidence [11] and will use the RepOrting standards for Systematic Evidence Synthesis (ROSES) [22] (Additional file 1).

### Search strategy

A search for primary literature, including peer-reviewed articles and gray literature will be performed using multiple indexing platforms, bibliographic databases, organizational websites, and other search platforms. There are no temporal constraints on the search. The geographic scope for the search is global because coral reef degradation and loss is a global issue [15]. Searches will be performed in English, and articles without a full text published in English will be documented and excluded. We made the decision to restrict the search to English language due to resource constraints and recognize that this introduces bias to the systematic map. Here, we describe details of the search strategy, including the search string development and literature search plan.

#### Search string development

The core team for the systematic map developed a list of keywords corresponding to the elements of the primary question (Table 1) for coral reefs (population), built structures (intervention), and both ecological and physical outcomes (outcomes) (Additional file 2). Search terms were combined into search strings (Table 2) and tested in Web of Science (Additional file 2). We developed five substrings, one of which narrowed the search to coral reefs. We originally tested the inclusion of the word reef* in the coral substring (coral* AND reef*), but multiple known articles that included relevant evidence did not mention the word reef in the title or abstract, so we retained only the word coral* in that substring. Two substrings describe the built structures and their context for use (intervention). Two additional substrings detail ecological and physical outcomes associated with the intervention.

**Table 2:** Search substrings created for population, interventions, and outcomes (PIO)

PIO criteria	Concept	Substring (web of science syntax)	Explanation
Population	Coral reefs	(coral*)	Substring for coral reefs
Intervention	Built structure	(artificial* OR gray* OR grey* OR engineer* OR hybrid* OR design* OR construct* OR install* OR built* OR build* OR deploy* OR sink* OR sunk* OR sank* OR modul* OR structur* OR biorock* OR concrete* OR "reef ball*" OR ecoreef* OR "eco reef*" OR "eco-reef*" OR "mars assisted reef restoration*" OR "mineral accretion*" OR tetrapod* OR tetrahedron* OR trapezoid* OR "reef mattress*" OR "reef unit*" OR "reef star*" OR "reef spider*" OR print* OR fabricat* OR rebar* OR artwork* OR sculpt* OR monument* OR decommission* OR ship* OR pipe* OR tire* OR tyre* OR bridge* OR repurpose* OR "re-purpose*" OR eternal* OR "self-healing*" OR "self healing*" OR terracotta* OR clay* OR ceramic* OR tile* OR "human-made*" OR "human made*" OR "man-made*" OR "man made*" OR manmade* OR biomimic* OR mimic* OR "biogenic structure*" OR "biogenic material*" OR limestone* OR boulder* OR rubble* OR cobble* OR rock* OR unconsolidate* OR "natural material*" OR "natural structure*" OR "natural reef*" OR "nature based solution*" OR "nature based strateg*" OR "nature based defen$e*" OR "nature based protection*" OR "nature based coastal" OR "nature based shoreline*" OR "nature based mitigation" OR infrastructure* OR "nature based infrastructure" OR "hybrid infrastructure" OR "hybrid technique*" OR "natural climate solution*" OR "natural infrastructure" OR "eco* engineer*" OR "eco-engineer*" OR ecoengineer* OR "eco* friendly engineering" OR "ecosystem friendly engineering" OR bioengineer* OR "blue engineering" OR "green engineering" OR "building with nature" OR "engineering with nature" OR "working with nature" OR "nature derived solution*" OR "nature based feature*" OR "nature inspired solution*" OR "nature inclusive design*" OR "nature inspired design*" OR "nature derived design*" OR "ecosystem* based adaptation*" OR "ecosystem* based mitigation" OR "disaster risk reduction" OR "coastal defen$e*" OR "blue infrastructure" OR "green infrastructure" OR "ecosystem based disaster risk reduction" OR "hazard* mitigation*" OR "hazard* risk*" OR "coast* protect*" OR "reefense" OR "x-reef*" OR stabili$*)	substring for built structures used for an intervention
Intervention	Context for intervention	(restor* OR mitig* OR enhanc* OR creat* OR supplement* OR rehabilitat* OR protect* OR "damage reduc*" OR "risk reduc*" OR attenuat* OR "coastal defen$e*" OR stabili$* OR recover* OR resilienc* OR "hazard risk*" OR conserv* OR infrastructure* OR "nature based*" OR engineer* OR recreat* OR touris* OR dredg* OR ground* OR "blast fish*" OR mining*)	Substring for goal (or reason) behind built structure intervention
Outcome	Ecological outcome	(grow* OR cover* OR communit* OR rich* OR divers* OR surviv* OR settle* OR dens* OR recruit* OR abund* OR size* OR coloniz* OR rugos* OR complexit* OR "surface area*" OR volume* OR connectiv* OR dispers* OR disease* OR mortalit* OR fragment* OR breakage* OR condition* OR bleach* OR succession* OR bioaccumul* OR "bio-accumul*" OR "chemical concentrat*" OR "biological interact*" OR succession* OR competit* OR predat* OR mutual* OR commensal* OR facilitat* OR parasit* OR omniv* OR zooplank* OR herbiv* OR piscivor* OR invasiv* OR invad* OR calcific* OR skelet* OR accret* OR gene OR genes OR genetic* OR corridor* OR distribut* OR composit* OR tissue* OR extens* OR zooxanth* OR symbio* OR microb* OR microorgan* OR "micro organ*" OR physiol* OR respir* OR photosynth* OR photopigm* OR histol* OR metabol* OR friction* OR bathy* OR curv* OR aspect* OR slop* OR fertiliz* OR embryo* OR planulat* OR health* OR diamet* OR "coral watch" OR stabili$* OR struct*)	Substring for ecological outcomes of built structure intervention
Outcome	Physical outcome	(wave* OR current* OR friction* OR rough* OR flood* OR inundat* OR protect* OR forc* OR eros* OR erod* OR "storm surge*" OR break* OR sediment* OR attenuat* OR energ* OR flux* OR reduc* OR mitig* OR defen* OR tide* OR tidal* OR "sea level*" OR "water level*" OR elevat* OR shoreline* OR scour* OR damp* OR amplif* OR expos* OR circulat* OR fetch* OR buffer* OR stress* OR velocit* OR speed* OR direction* OR magnitud* OR redistribut* OR compact* OR consolid* OR trap* OR retain* OR retent*)	Substring for physical outcomes of built structure intervention

Substrings are in Web of Science syntax

The six search substrings will be combined into one search string as follows:

**Table:** 

Population: (coral reef substring)
**AND**
Intervention: (built structure substring AND context for built structure intervention substring)
**AND**
Outcome: (ecological outcome substring **OR** physical outcome substring)

The combined search string will be used to search titles and abstracts. More specifically, search results will be those where search string terms appear in the title, in the abstract, or in both the title and abstract (e.g., search syntax will be title OR abstract).).

#### Searching the literature

Searches for relevant primary literature will be performed in indexing platforms, bibliographic databases, open discovery citation indexes, and a web-based search engine (Table 3). Search strings were originally developed using Web of Science search syntax thus will be modified as needed to meet syntax requirements of other indexing platforms and bibliographic databases. The web-engine search will be performed using Google Scholar via Harzing’s Publish or Perish Software [23]. The search string used for Google Scholar will be adapted to meet syntax limitations of the platform, will be performed on title only, and will be restricted to the first 1000 results [21]. Searches will also be performed using Inciteful (https://inciteful.xyz/), a novel literature discovery tool, for up to the first 1000 similar results [57]. Specifically, the search will be seeded with benchmarking articles; no search string is required for Inciteful.

**Table 3:** List of sources to be searched for relevant primary literature

Source type	Source name	Indexes	Search filters	Subscription	Provider
Indexing platform	Web of Science Core Collection (WoS)	– SCI-expanded (1980-present) – SSCI (1980-present) – CPCI-S (1990-present) – CPCI-SSH (1990-present) – ESCI (2018-present)	Document type: Article, Proceedings Paper, Early Access, Data paper	Duke University	Clarivate
Indexing platform	Scopus	Scopus		Duke University	Elsevier
Bibliographic databases	ProQuest Earth, Atmospheric, & Aquatic Sciences Collection	– Aquatic Sciences and Fisheries Abstracts – Meteorological and Geoastrophysical Abstracts – Earth, Atmospheric, & Aquatic Sciences Database – Oceanic Abstracts	Source type: Scholarly Journals, Dissertations & Theses, Conference Papers & Proceedings, Reports	Duke University	ProQuest
Open discovery citation index	LENS.org	– CORE – Crossref – PubMed – Microsoft Academic	Document type: Journal Article, Conference Proceeding Article, Conference Proceedings, Dissertation, Report	N/A	Cambia
Open discovery citation index	Dimensions	N/A	Publication type: Article, Proceedings	N/A	Digital Science
Web-based search engine	Google Scholar via Harzing’s Publish or Perish	Google Scholar	Title search Up to the first 1000 results Search string will be adapted to fit Google Scholar	N/A	Google via Publish or Perish [23]
Novel literature discovery tool	Inciteful	N/A	Seeded with benchmarking articles Up to first 1000 results No search string required	N/A	Weishuhn [57]

The source indexes and filters are stated, as well as applicable search filters

Twenty organizational websites will also be searched for evidence (Table 4). The organizations span government agencies, nonprofit organizations, and other entities that report on the use of built structures in coral reef ecosystems. Most organizational websites do not permit Boolean searches, so search strings will be by hand and details of how searches were implemented will be documented. Gray literature will be screened in situ, and up to 100 results per organizational website will be screened.

**Table 4:** Names of organizational websites to be searched for evidence

Organization name	URL
Conservation International	https://www.conservation.org/
Coral Reef Alliance	https://coral.org/en/
Florida Department of Environmental Protection	https://floridadep.gov/
Global Coral Reef Alliance	https://www.globalcoral.org/
International Union for Conservation of Nature	https://www.iucn.org/
National Oceanic and Atmospheric Administration	https://www.noaa.gov/
Sea Grant	https://seagrant.noaa.gov/
Reef Base	http://reefbase.org/
The Nature Conservancy	https://www.nature.org/
United Nations Decade on Restoration	https://www.decadeonrestoration.org/
United Nations Development Programme	https://www.undp.org/
United Nations Environment Programme	https://www.unep.org/
United Nations Environment Programme World Conservation Monitoring Center	https://resources.unep-wcmc.org/
U.S. Army Corps of Engineers	https://www.usace.army.mil/
U.S. Geological Survey	https://www.usgs.gov/
U.S. Fish and Wildlife Service	https://www.fws.gov/
Wildlife Conservation Society	https://library.wcs.org/
World Bank	https://www.worldbank.org/
World Resources Institute	https://www.wri.org/
World Wildlife Fund	https://www.worldwildlife.org/

Each organization’s name and URL are provided

### Comprehensiveness of the search

The evidence synthesis team identified 21 benchmarking articles to test against the search string (Additional file 3). These benchmarking articles were sourced from subject matter experts, including those from the core research team. Search strings were tested in Web of Science, and 18 of the 21 articles were indexed in Web of Science (e.g., 3 articles were not indexed in Web of Science meaning that they are not part of the Web of Science collection and so will not be found in Web of Science regardless of the search string used). Search strings were adjusted incrementally until all but two of the 18 indexed articles were identified. The two articles that were unable to be identified in Web of Science did not include terms related to the intervention in the title or abstract. These articles had been provided by the synthesis team because they had case studies embedded within that used built structures but were deemed undetectable in the search since the intervention was not covered in the title and abstract.

### Reference management and deduplication

Reference management will be conducted using Clarviate’s EndNote (version 20) citation management software [46]. RIS files from searches implemented on different platforms (e.g., indexing platforms, bibliographic databases) will be uploaded separately to EndNote and references deduplicated using built-in EndNote functions and open-source tools, such as the R package ‘CiteSource’ [40]. Reference metadata will be checked and fixed as needed. Cleaned references will be combined into one.RIS file and uploaded to the title and abstract screening software, Swift-Active Screener (Sciome LLC; [26], for review. Following review of title and abstracts, updated.RIS files of included and excluded articles will be exported from the screening software. The .RIS file corresponding to articles that passed title and abstract screening will then be imported to EndNote for full text screening. Screeners will use EndNote to review references during full text screening and will track reference inclusion and conduct metadata coding using Google spreadsheets. RIS records of included and excluded articles will be kept for ROSES reporting.

## Article screening and study eligibility criteria

### Screening process

Articles returned from literature searches will be screened against eligibility criteria in two stages, first by title and abstract and second by full text (Table 5). The software Swift-Active Screener will be used for title and abstract screening because it utilizes a combination of screener feedback and a type of machine learning termed active learning [26]. The active learning algorithm incorporates screener feedback on which articles are deemed relevant or irrelevant. Specifically, the algorithm ranks and shuffles unscreened articles so that articles it designates as relevant can be prioritized for screening. Screening will occur until the software’s “recall rate” reaches 95% [26]. The “recall rate” is the running estimate of the percentage of relevant references that have been screened from the original set. Previous studies have demonstrated that Swift Active Screener, with its active learning algorithm and ranking system, can provide significant time resource savings. Use of Swift Active Screener is growing within environmental sciences [56] and is accepted in medical sciences [13, 20]. Using Swift Active Screener may introduce some bias into the systematic map if articles are overlooked as a product of the algorithm and ranking system; however, we expect ~ 20,000 results, making the use of Swift Active Screener necessary and helpful. Keywords will be highlighted in Swift to help identify information relevant for assessing eligibility criteria. Screeners will indicate in Swift whether articles should be included or excluded based on the eligibility criteria. Articles that pass title and abstract screening will be screened at the full text stage to determine whether they still meet eligibility criteria and should be included in the study or not. If the full text for an article cannot be located, the article will be excluded. Exclusion rationale will be documented during both screening stages.

**Table 5:** Preliminary eligibility criteria for literature on built structures used for coral restoration

Criteria	Overview	Included	Excluded
Population of subjects	Shallow, tropical coral reefs	Coral reefs in nearshore, shallow water depths (≤ 30 m) in tropical latitudes (35° N to 35° S) where built structure interventions occur Coral reefs created by or facilitated by a built structure intervention in a location devoid of reefs (e.g., intervention on soft sediment that creates or is intended to create reef) can also be included	Coral reefs in cold, deep (> 30 m), or mesophotic waters are excluded. Coral reefs > 35° N or > 35° S are excluded. Other types of reefs, like rocky reefs, are excluded. All other coastal, marine, terrestrial, freshwater, or subterranean systems are excluded
Intervention	Built structure established in coral reef systems	Interventions must use a built structure. Built structures may include those of: 1. Artificial or human-made origin, including structures engineered or designed for reef contexts with or without electricity, structures repurposed from their primary use, and those structures created as artwork 2. Hybrid origin that are created from a combination of artificial and natural material, such as cement plus natural rock 3. Natural origin from geologic sources, such as mined rock, limestone, or boulders These interventions can be related to coral reef-related applications, including restoration, remediation, mitigation, enhancement, rehabilitation, rebuilding, stabilization, providing coastal protection or defense, tourism and recreation, research, etc. These interventions can be established in response to general habitat degradation and chronic disturbances or in response to pulse disturbances, like storms, blast fishing, dredging, mining, and ship groundings Interventions using electrification and a built structure will be included	Interventions without built structures
Comparator	Comparator	No comparator is required because the only requirement is the presence of built structure Studies that include a comparator, however, will also be included. Comparators may include: presence vs. absence of built structure intervention, before vs. after built structure intervention, different types of built structure interventions, different projects or sites with the same built structure intervention type, different reef types (e.g., built structure on fore- vs. back-reef), built structure vs. natural coral reef	N/A—no comparator required
Outcome	Ecological and physical performance outcomes	Ecological and physical performance outcomes of built structure interventions that are measured, observed, or modeled Ecological outcomes must relate to coral and coral reef metrics, such as recruitment, growth, morality, condition, rugosity, and cover (Table 7). Ecological metrics related to biological interactions with coral will be included Physical outcomes must relate to waves, currents, erosion, flooding, and other coastal processes (Table 8) Performance outcomes may be related to the built structure or adjacent areas. For example, ecological outcomes like coral growing on the built structure or coral growing adjacent to the built structure would both be included	Ecological outcomes solely related to other trophic groups, such as non-coral invertebrates, macroalgae, and fish. Broader ecosystem-level metrics or processes, such as productivity, pollution, and nutrient cycling. Chemical, economic, and social outcomes
Study type	Experimental, observational, or modeling studies	Experimental, quasi-experimental, modeling (statistical, theoretical), or observational studies with quantitative data. Field and lab studies are included	Reviews, meta-analyses, theoretical studies, commentaries, editorials, opinions, or perspectives

Details are provided for the population, intervention, comparator, outcome, and study type criteria

Screeners will be trained on how to reproducibly conduct both screening stages. Training will occur in dedicated training sessions where select articles are screened as a group before select additional articles are screened individually. Inconsistencies in screening decisions will be discussed and used to refine eligibility criteria. Once screeners are trained, quantitative assessments of inter-reviewer consistency will be conducted by generating Kappa statistics or percentage agreement values for all pairs of reviewers for a set of 100 randomly selected titles and abstracts. Double screening will be conducted for up to 5% of the title and abstract or full text screening stages. Single screening may introduce bias into the systematic map, but because of resource constraints and the high number of expected articles (~ 20,000), it is necessary. With the expected number of articles, 5% would be ~ 1000 articles. Screeners cannot screen articles for which they were an author or coauthor.

### Eligibility criteria

Eligibility criteria include the population of subjects, intervention, comparator, outcome, and study type (Table 5).

### Relevant population(s)

The relevant population for this systematic map is coral reefs located in nearshore, shallow tropical waters. We define shallow as ≤ 30 m. We define tropical waters as those between 35° N and 35° S latitude; this may include some water typically designated as subtropical depending on the latitudinal classification scheme. Reef types may include barrier reefs, reef flats, fringing reefs, reef crests, patch reefs, reef complexes, bommies, or atolls, as well as locations like forereef and backreef. If a coral reef is created by a built structure intervention (e.g., built structure led to (or was intended for) coral reef creation on a former soft sedimentary seascape, such as one composed of carbonate sand), then it is also eligible for inclusion. Coral reefs located in deep waters or mesophotic zones are excluded. Reefs with substrate other than carbonate deposited by coral, such as rocky reefs or sponge reefs, are also excluded. All other marine, coastal, terrestrial, freshwater, and subterranean ecosystems are also excluded.

#### Relevant intervention(s)

Interventions of interest for the systematic map are those that establish built structures in shallow, tropical coral reef settings (Table 6). Here, we define built structures as those that have been engineered, designed, created, built, or constructed using artificial, hybrid, or natural materials. Structures that use a combination of artificial and natural materials, classified as hybrid, are included. Structures such as natural rock or boulders are also included, so long as they are used as part of an intentional, active intervention because using mined rock or other naturally sourced boulders, rubble, or rock can be conducted in mitigation and restoration contexts. Eligible structures span a spectrum of those that were intentionally designed using cutting edge technologies (i.e., 3D printing) and engineering processes (design analyses for performance and design optimization), those designed using simpler approaches such as concrete castings, those that were created as artwork (i.e., underwater sculptures), and those that were repurposed for reef systems (i.e., natural rocks, repurposed concrete). Built structures must have been placed in nearshore, shallow environments suitable for coral reefs and may include those with varied goals. For example, goals or the context of the built structure intervention may include reef creation, enhancement, remediation, rehabilitation, or stabilization, including to address impacts like ship grounding, blast fishing, coral mining, dredging, storm damage, bleaching events, invasive species, or other damages. Goals can also include offsetting damages to coral reefs in other areas or more simply testing the effectiveness of built structures for coral restoration, as well as tourism and recreation or research related goals.

**Table 6:** Typology of built structures interventions. Typologies adapted from [2, 4, 5, 8, 25, 43]

Category	Definition	Examples
Artificial—designed	Artificial or human-made structures that have been designed or engineered specifically for reef contexts and do not contain electricity	Reef modules; reef balls; caste concrete; reef mattresses; reef units; reef castles; concrete modules; Mars Assisted Reef Restoration System (MARRS reef stars); biorock without electricity; tetrapods; Subcon reef modules; 3D frames; Autonomous Reef Monitoring Structures (ARMS)
Artificial—designed with electricity	Artificial or human-made structures that have been designed or engineered specifically for reef contexts and do contain electricity	Biorock with electricity; seacrete; mineral accretion technology; 3D frames with electricity; any module with electricity
Artificial—repurposed	Artificial or human-made structures that have been repurposed from their primary use	Concrete pipes; concrete rubble; ships; tires; planes
Artificial—artwork	Artificial or human-made structures that have been created as artwork	Underwater sculptures; underwater statues; underwater art installation (ex: 1000 Mermaids Artificial Reef Project)
Hybrid—artificial and natural	Hybrid structures, defined as those created from a combination of artificial and natural materials	Cement plus natural rock; mesh or netting over existing rubble; gabion cages; gabion baskets; reef bags
Natural	Natural structures of geologic origin	Cobble; rock; boulder; limestone; unconsolidated reef

#### Relevant comparator(s)

No comparator is necessary for an article to be eligible. It must only contain the intervention. If a study does include a comparator, though, it is eligible, and the comparator type will be recorded. Potential comparators include locations with built structures versus without; before, during, or after built structure intervention; different types of built structure interventions.

#### Relevant outcome(s)

Outcomes from built structure interventions in coral reef settings include ecological and physical performance categories. Specific typologies were developed to characterize these ecological and physical performance outcomes (Tables 7; 8;). Briefly, ecological performance categories relate specifically to coral and coral reef metrics, such as recruitment, growth, morality, condition, rugosity, and cover (Table 7). Ecological metrics related to biological interactions with coral are included, but outcomes solely related to other trophic groups, such as other non-coral invertebrates, macroalgae, and fish are not the focus of the systematic map thus are excluded. Broader ecosystem-level metrics or processes, such as productivity, pollution, and nutrient cycling are also outside the scope of this systematic map. Physical performance categories of interest relate to waves, currents, erosion, flooding, and other coastal processes related to coastal protection and coastal resilience (Table 8). Chemical, social, and economic outcomes are beyond the scope of the map.

**Table 7:** Typology of ecological performance outcomes. Typologies are largely adapted from [35], and to a lesser extent from [7, 9, 42, 56]

Category	Definition	Examples
Bioaccumulation	Outcomes focused on characteristics related to bioaccumulation of chemicals in coral	Chemical concentration in coral
Biological interactions	Outcomes focused on characteristics of or changes in biological or species interactions like facilitation, competition, predation	Competition; predation; mutualism; commensalism; facilitation; herbivory; omnivory; zooplanktivory; invasive or non-native species interactions with other organisms; succession
Calcification	Outcomes focused on characteristics related to calcification of coral	Calcification rate; skeletal structure; skeletal density; accretion
Condition	Outcomes focused on characteristics related to the physical condition of coral, including bleaching and disease	Coral condition; fragmentation; breakage; color change; bleaching rate; bleaching frequency or proportion; amount bleached; bleaching prevalence; disease frequency; disease incidence; disease prevalence and occurrence
Connectivity	Outcomes focused on characteristics related to connectivity of coral at any scale (colony, population, system)	Population connectivity; genetic connectivity; metapopulation connectivity; ecosystem connectivity; dispersal; habitat connectivity; habitat corridors
Cover	Outcomes focused on characteristics related to cover of coral	Coral cover; percent cover
Distribution	Outcomes focused on characteristics related to the spatial distribution of coral	Spatial distribution; spatial occurrence; spatial presence / absence
Diversity	Outcomes focused on characteristics related to community metrics or diversity of coral	Taxa richness; diversity index; species richness; species diversity; taxa composition; community composition
Genetic	Outcomes focused on characteristics related to genetics of coral	Gene expression; population genetic structure
Growth	Outcomes focused on characteristics related to growth of coral	Skeletal growth; tissue growth; linear extension
Microbiome	Outcomes focused on characteristics related to the microbiome associated with coral	Density and characteristics of Symbiodiniaceae and other coral-associated microorganisms (zooxanthellae, other microorganisms in mucus, etc.)
Mortality	Outcomes focused on characteristics related to mortality (including survival) of coral	Mortality; survival; larval mortality; larval survival
Physiology	Outcomes focused on characteristics related to physiology of coral	Content in carbohydrates, lipid, protein, or other biomolecule; enzyme activity; histology; metabolism; mucus production; photopigment concentration; photosynthesis efficiency; respirate rates
Recruitment	Outcomes focused on characteristics related to recruitment and settlement of coral	Settlement success; number of recruits; settlement metrics; colonization
Reef structure	Outcomes focused on characteristics related to reef structure formed by coral	Rugosity; structural complexity; surface area; volume; roughness; friction factor; reef height; vertical relief; slope; aspect; bathymetry; curvature
Reproduction	Outcomes focused on characteristics related to reproduction of coral	Fertilization success; embryo development; state of reproductive structure; planulation
Species or population	Outcomes focused on characteristics of species or populations	Abundance; density; size; size structure; size classes; community structure

The ecological outcomes focus on characteristics of coral related to or affected by built structure interventions

**Table 8:** Typology of physical performance outcomes. Typologies are adapted the same as used in [56]. Typologies are adapted from [1, 6, 14, 45]

Category	Definition	Examples
Waves	Processes or characteristics related to waves, including their energy and height and whether waves are attenuated or amplified	Wave attenuation, dissipation, dampening; wave amplification or build up; wave height; wave length; wave period; wave energy; wave breaking; resistance to waves; wave diffraction; wave breaker or breaking; duration; wave exposure; wave orbital velocity; wave runup; wave celerity; wave direction; wave frequency; wave setup; wave steepness
Currents	Processes or characteristics related to currents, including magnitude, direction, and exposure	Speed; velocity; turbulence; direction; exposure; current patterns; circulation
Wind	Processes or characteristics related to wind, including speed, fetch, and buffering	Speed; fetch; direction; buffering; exposure; wind setup; wind stress
Water level	Processes and characteristics of water level, including flooding and inundation and sea level rise	Flood or inundation height; flood or inundation level; flood or inundation frequency; flood or inundation duration; inundation tolerance; king tide; tides; tidal range; mean sea level; water level
Storm surge	Processes or characteristics related to storm surge, including storm surge height and velocity, as well as storm surge attenuation	Storm surge attenuation or dissipation; storm surge amplification or propagation; storm surge height, elevation, levels, velocity, rates, timing; water storage; resistance to storm surge; surge-attenuation rate
Sediment and morphology	Processes or characteristics related to sediment and morphology, including erosion and accretion and shoreline change	Sediment deposition; sediment accretion; sediment loss; sediment redistribution; sediment compaction; sediment consolidation; sediment resuspension, sediment flux; elevation buildup; sediment stability; sediment trapping; shoreline change rate; shoreline morphology; shoreline change; elevation change; sediment retention; scour

The physical outcomes focus on characteristics of the physical environment related to or affected by built structure interventions. Forces, flux, and energy fall within specific categories; for example, wave forcing would fall within the wave category

#### Relevant study type(s)

Studies that report quantitative data from observational, experimental, quasi-experimental, modeling, or simulation studies are included. Studies conducted in situ (field) or ex situ (lab, flume, etc.) or in computing environments (modeling, simulation) will be included. If studies are reviews, meta-analyses, theoretical, opinions, perspectives, or commentaries, they are ineligible because they do not report quantitative findings.

### Study validity assessment

Study validity will not be systematically assessed because this is a systematic map which aims to collate and summarize the distribution and abundance of evidence. During data coding, attributes will be extracted that can be used for follow-up assessments of study validity for subsets of the evidence base.

## Data extraction and coding strategy

Metadata attributes from studies that adhere to eligibility criteria will be entered into a data “coding” spreadsheet (Table 9; Additional file 4). The attributes will include bibliographic information, as well as those related to the population, intervention, study type, comparator, and outcome. For example, intervention attributes will include the type of built structure intervention (Table 6), the structure material, proprietary name (if applicable), policy-relevant term, and description of the coral restoration context. Details on each metadata attribute are provided in a code book adapted from a code book used in [56] *.* The code book provides a description of each attribute, instructions for data entry, and levels of categorical attributes that screeners can select from dropdown menus. We do not plan to contact authors to request missing information. Rather, if the required information is not stated in the article, it will be coded as “unknown.” If an attribute is not applicable to an article, the attribute will be coded as “not applicable.”

**Table 9:** Metadata attributes planned for extraction during data coding

Category	Attribute name	Description
Bibliographic	Publication type	Type of publication [peer-reviewed, book chapter, etc.]
	Author(s)	Article author(s)
	Publication year	Year article was published [YYYY]
	Title	Title of article
	Journal name	Name of journal where article was published
	Volume	Volume of journal in which article was published
	Page numbers	Page numbers of article
	DOI	DOI of article
	URL	URL of article
Population	Population eligibility	Inclusion versus exclusion decision during full text screening stage for population [include, exclude]
	Reef type	Type of coral reef [atoll, fringing, barrier, etc.]
	Reef relief	Vertical relief of reef [high, low]
	Reef location	Location on reef where study occurred [fore reef, back reef, reef crest]
	Reef energy regime	Categorical classification of reef energetic environment [high energy, low energy]
	Description of coral reef	Description from article of coral reef
Intervention	Intervention eligibility	Inclusion versus exclusion decision during full text screening stage for intervention [include, exclude]
	Category of built structure intervention	Category of built structure intervention [artificial—engineered, artificial—repurposed, hybrid, natural, etc.] [Table 6]
	Description of built structure intervention	Description from article of built structure intervention including quantitative or qualitative information like structure type and materials, structure size, structure date, deployment setting, etc
	Material of built structure	Material of built structure [metal, concrete, rock, wood, plastic, 3D printed, etc.]
	Proprietary name of built structure	If applicable, the proprietary name of built structure [Reef Balls, MARRS Spider, etc.]
	Policy-relevant term for intervention	Policy-relevant term used to describe built structure intervention [nature-based solution, artificial reef, remediation structure]
	Depth of built structure	Depth [mean or range, if available] of built structure location
	Dates of built structure intervention	When the built structure intervention takes place
	Cost of built structure intervention reported	Whether cost information on built structure intervention is reported [yes, no]
	Coral restoration or related context of intervention	Whether the built structure intervention relates to coral restoration or related applications [coral restoration context; environmental mitigation context; coastal defense context]
	Description of how intervention relates to coral restoration or related contexts	Description from article of how built structure relates to coral restoration or similar contexts
Study type	Study type eligibility	Inclusion versus exclusion decision during full text screening stage for study type [include, exclude]
	Study type	Type of study [experimental, modeling, etc.]
	Study objective	Description from article of study objective
	Study design	Description from article of study design
	Study location	Description from article of study location
	Geographic scale	Description from article of built structure intervention geographic scale [global, regional, national, subnational, local]
	Country	Country where built structure intervention occurred
	State	If built structure installed in United States, state where built structure intervention occurred
	Water body	Name of water body where study was conducted
	Category of comparator	Category of comparator used in study [before vs. after, presence vs. absence, different types of built structure interventions, etc.]
	Description of comparator	Description from article of study comparator, if applicable
Outcome	Outcome eligibility	Inclusion versus exclusion decision during full text screening stage for outcome [include, exclude]
	Category of outcome	Whether performance outcome is ecological or physical [ecological or physical]
	Subcategory of outcome	Subcategory of outcome [ecological: coral cover, coral condition, etc.]; see outcome typology [Tables 7, 8]
	Description of outcome	Description from article of outcome
	When outcome evaluation took place	Whether built structure outcomes were evaluated before, during, or after construction [before construction, during construction, after construction (≤ 1 yr), after construction (> 1 to ≤ 5 yrs), after construction (> 5 to ≤ 10 yrs), after construction (> 10 yrs), no evaluations conducted]
	Frequency of outcome evaluation	Frequency of outcome performance evaluation, including units (e.g., every 3 weeks for 5 years)
	Methods for outcome evaluation	Monitoring method(s) used to evaluate built structure performance outcome [net sampling, economic survey, etc.]
	Metrics for outcome evaluation	Monitoring metric(s) used to evaluate built structure performance outcome [coral growth, coral mortality, etc.]
	Data type for outcome evaluation	Whether data are qualitative, quantitative, or a combination of both to monitor performance outcome
	Directionality of outcome	Directionality of measured performance outcome [positive, negative, mixed, neutral / no effect]
Additional	Notes	Notes on data extraction

These attributes will be extracted from articles that pass title and abstract screening, as well as full text screening. Attributes are categorized to encompass bibliographic information, population information, intervention information, etc. Outcome attributes, such as outcome category and subcategory, outcome description, etc. will be repeated for each outcome (e.g., coral condition, waves). Additional details provided in Additional file 4

Screeners will be trained to code metadata reproducibility during a training session. The training session will focus on collectively coding data for several articles. Each screener will then be assigned a subset of articles to code independently. Coding results will be compared qualitatively and the group will discuss inconsistencies and alter attributes and instructions if necessary. Double data extraction, which is the extraction of data from a study by multiple screeners, will not be conducted because of the high number of anticipated articles that will require data coding. Instead, we will conduct spot checks for a percentage of articles. The percentage of articles that we spot check in the systematic map will be reported.

## Study mapping and presentation

Metadata from studies that meet eligibility criteria at both the title and abstract and full text screening stages will be analyzed to identify patterns in the distribution and abundance of evidence related to the use of built structures in coral restoration and related applications. Analyses will be conducted in R [36] to answer the posed primary and secondary research questions, characterize the evidence base, and identify both evidence clusters and evidence gaps. Specifically, the extent of evidence on different types of built structure interventions by their typology (Table 6), material, proprietary name, and policy relevant term will be characterized. Similarities and differences in the evidence base according to the context that the built structure intervention was intended, such as coral restoration, environmental mitigation, and coastal protection, will be identified. The abundance and distribution of evidence across ecological and physical outcomes for which built structures have been evaluated, as well as for study setting—geographic region, spatial scale, and seascape environment—will be cataloged. When feasible, the directionality of evidence (e.g., positive, negative, neutral) will be documented. Evidence clusters and gaps will be identified with heat maps displaying the number of studies corresponding to cross-tabulated attributes.

Findings will be compiled into an evidence map for peer-reviewed publication that will include a narrative summary of the evidence base. This state of the science review will be complemented by visual depictions of the evidence base using heat maps, bar plots, and geographic distribution maps. Tabular summaries of findings may also be included. The systematic map will emphasize the discovery of evidence clusters and gaps, and suggest potential avenues for future research. Map findings may be applied to help improve practice and help inform policy and management decisions regarding the potential use of built structures in tropical, shallow coral reefs. Map findings will also inform systematic reviews on the quantitative effectiveness of built structures. All data on included and excluded literature and associated metadata will be made publicly available.

## Supplementary Information

**Additional file 1. **ROSES for systematic map protocols checklist.

**Additional file 2. **Search strategy development and testing.

**Additional file 3. **Benchmarking articles.

**Additional file 4. **Data extraction codebook.

## Data Availability

Not applicable.
